# Prognostic Significance of Estrogen Receptor Alpha in Oral Squamous Cell Carcinoma

**DOI:** 10.3390/cancers13225763

**Published:** 2021-11-17

**Authors:** Christian Doll, Carolin Bestendonk, Kilian Kreutzer, Konrad Neumann, Anne Pohrt, Irena Trzpis, Steffen Koerdt, Steffen Dommerich, Max Heiland, Jan-Dirk Raguse, Korinna Jöhrens

**Affiliations:** 1 Department of Oral and Maxillofacial Surgery, Charité—Universitatsmedizin Berlin, Corporate Member of Freie Universitat Berlin and Humboldt-Universitat zu Berlin, Augustenburger Platz 1, 13353 Berlin, Germany; carolin.bestendonk@charite.de (C.B.); kilian.kreutzer@charite.de (K.K.); irena.trzpis@charite.de (I.T.); steffen.koerdt@charite.de (S.K.); max.heiland@charite.de (M.H.); 2 Institute of Biometry and Clinical Epidemiology, Charité—Universitatsmedizin Berlin, Corporate Member of Freie Universitat Berlin and Humboldt-Universitat zu Berlin, Charitéplatz 1, 10117 Berlin, Germany; konrad.neumann@charite.de (K.N.); anne.pohrt@charite.de (A.P.); 3 Department of Otorhinolaryngology, Charité—Universitätsmedizin Berlin, Corporate Member of Freie Universitat Berlin and Humboldt-Universitat zu Berlin, Charitéplatz 1, 10117 Berlin, Germany; steffen.dommerich@charite.de; 4 Department of Oral and Maxillofacial Surgery, Fachklinik Hornheide, 48157 Münster, Germany; Jan-Dirk.Raguse@fachklinik-hornheide.de; 5 Institute of Pathology, Technische Universität Dresden, 01307 Dresden, Germany; korinna.joehrens@uniklinikum-dresden.de

**Keywords:** oral squamous cell carcinoma, estrogen receptor alpha, survival, prognosis, hormone receptor

## Abstract

**Simple Summary:**

Although the survival rate has improved over the past decades, the prognosis of oral squamous cell carcinoma (OSCC) is still poor, and new treatment strategies are required. The aim of this study was to evaluate estrogen receptor alpha (ERα) expression in OSCC in a large patient cohort as a potential prognostic marker and therapeutic target. The findings indicated a rare expression of ERα that, however, was associated with a dramatic decrease of overall survival in male patients. In ERα-positive OSCC patients, an ER-based therapeutic (adjuvant) approach in the future might be conceivable based on the findings of this study.

**Abstract:**

Introduction: Several studies suggest an estrogen receptor alpha (ERα)-mediated influence on the pathogenesis of oral squamous cell carcinoma (OSCC), as described for other malignancies that are not considered to be primarily hormone-dependent. Recently, an association between ERα expression and improved survival in oropharyngeal squamous cell carcinoma (OPSCC) has been found. However, the prognostic relevance of ERα in OSCC has not been proven to date. Therefore, the aim of this study was to evaluate ERα expression in OSCC in a large patient cohort and analyze its influence on survival and recurrence. Material and Methods: A total of 316 patients with primary OSCC who received initial surgical therapy were included in this analysis. The expression of ERα was evaluated on tissue microarrays by immunohistochemistry in the primary tumor and/or primary lymph node metastases. The expression level was quantified by light microscopy using the immunoreactive score (IRS) for estrogen receptor detection. An IRS equal to or greater than 2 was considered positive. The 5-year overall survival (OS) and relapse-free survival (RFS) were examined by the Kaplan–Meier method and log-rank test. Results: A total of 316 patients (111 females; 205 males) with a mean age of 61.3 years (range 27–96 years) were included in this study. In 16 patients (5.1%; 6 females and 10 males), positive ERα expression was found in the primary tumor (*n* = 11; 11/302) or lymph node metastases (*n* = 5; 5/52). Patients with positive ERα expression in primary tumors/primary lymph node metastases had a significantly lower OS and RFS (*p =* 0.012; *p* = 0.0053) compared to ERα-negative patients. Sub-group analysis in relation to gender revealed a highly significant influence of ERα expression on OS and RFS in males but not in females, both for the ERα-positive primary tumor cohort (males: *p* = 0.0013; *p* < 0.0001; females: *p* = 0.56; *p* = 0.89) and the ERα-positive primary tumor/primary lymph node metastasis cohort (males: *p* < 0.0001; *p* < 0.0001; females: *p* = 0.95; *p* = 0.96). In multivariate cox regression analysis, the ERα IRS of primary tumors (dichotomized; ERα+ vs. ERα−) was an independent risk factor for OS (HR = 4.230; 95%CI 1.616–11.076; *p* = 0.003) and RFS (HR = 12.390; 95%CI 4.073–37.693; *p* < 0.001) in the male cohort. There was a significant difference (*p* = 0.006) of ERα positivity with regard to the localization of the primary tumor. ERα positivity in the primary tumor was significantly associated (*p* = 0.026) with UICC stage, with most of the cases being diagnosed in stage IV. Furthermore, there was a significantly (*p* = 0.049) higher rate of bone infiltration in ERα-positive patients. Conclusion: Expression of ERα is rare in OSCC; however, it is associated with a dramatic decrease in OS in male patients. Further studies are necessary to confirm our results and to evaluate the exact mechanism underlying this observation. Hence, ERα-positive OSCC patients might benefit from an ER-based therapeutic (adjuvant) approach in the future.

## 1. Introduction

Oral squamous cell carcinoma (OSCC) is the most frequent malignancy affecting the oral cavity, with an annual incidence of more than 300,000 new cases worldwide [1]. The successful treatment of OSCC often requires a multimodal approach, and surgery is the therapeutic basis for most patients. Although survival has improved over the past decades [2], prognosis is still poor, and new treatment strategies are required.

Regular smoking and alcohol abuse are known to be typical risk factors for OSCC, also in young patients [3]. Despite these well-documented risk factors, the human papilloma virus (HPV) has been discussed as a potential cause, although it appears to play a minor role compared to that it performs in oropharyngeal squamous cell carcinoma (OPSCC) [4], which is another entity of the heterogenous group of head and neck squamous cell carcinoma. However, patients may develop OSCC without exposure to those common risk factors, which led to the conclusion that there is a pathological impact of other factors, which are not yet understood.

Estrogen receptors (ER) are involved in the regulation of many complex physiological processes in humans. Pathological ER signaling leads to the development of a variety of diseases, such as metabolic and cardiovascular disease, neurodegeneration, inflammation, osteoporosis, and cancer [5]. There are two different sub-types of estrogen receptors, which are widely distributed in different tissues, i.e., ER alpha (ERα) and ER beta (ERβ). Perturbation of the expression of these receptors has been found in many different cancers and provides a possible therapeutic target [6]. This has been successfully established in breast carcinoma [7]. However, ER-mediated treatment strategies are being investigated in many different tissues which are not all considered to be primarily hormone-dependent [5,8,9,10].

Various clinical observations suggest a hormone-dependent influence in the pathogenesis of OSCC. The highest incidence among female patients is seen after menopause. This might lead to the conclusion that a decrease of the estrogen level could play a role in cancer initiation [11,12]. Further observations such as an increasing incidence of OSCC in young non-smoking women within the last decades [13,14,15,16] or an increasing incidence of women with OSCC after hysterectomy also suggest that there is a hormonal influence on carcinogenesis [12]. Since the exposure to tobacco and/or alcohol can modify the level of testosterone and estrogen, a co-dependent hormonal impact on OSCC pathogenesis might be possible [11].

Several studies have discussed the influence of estrogen receptor (ER) on the pathogenesis of OSCC [17,18,19,20,21,22,23,24]. In OPSCC, which is another entity of the heterogenous group of head and neck cancers, an association between ERα expression and improved survival has recently been found [25]. A study published by Egloff et al. revealed an ERα-positivity of 95% in 56 evaluated head and neck tumors (including 23 OSCC); however, a concrete allocation only for OSCC was not mentioned [20]. This study shows a significantly reduced progression-free survival in patients with high (nuclear) ERα (and EGFR) tumor levels. However, a prognostic relevance of ER expression for OSCC alone has not been proven to date.

In general, information about the immunohistochemical expression of ERα in OSCC primary tissues is rare and remains controversial. The prevalence of ERα when evaluating (paraffin-embedded) OSCC tissue ranges from 0 to 13% [19,21,24,26,27,28].

To date, only small patient cohorts have been analyzed for ERα expression in OSCC, and no prognostic relevance has been found. Therefore, the aim of this study was to evaluate ERα expression in OSCC in a large patient cohort and analyze its influence on survival and recurrence.

## 2. Material and Methods

### 2.1. Ethics Statement

The Ethics Committee of the Faculty of Medicine Charité Berlin approved this study (EA2/028/15).

### 2.2. Patients

In this study, 316 patients with primary OSCC treated at Charité–Universitätsmedizin Berlin between 2005 and 2011 were included. Tumor staging was performed using the 7th edition of the American Joint Committee on Cancer (AJCC) Staging Manual [29], and treatment as well as follow-up were performed according to the current national guidelines at the time of presentation. Only patients who received initial surgical therapy were included in this study. The medical records of all patients were reviewed retrospectively.

Follow-up time for overall survival (OS) was defined as the time from the date of the first diagnosis until death. Patients without an event were censored at last contact or at the end of the observation period if the patient was still alive. In cases in which a date of primary diagnosis was not available (e.g., external diagnosis), the date of the first diagnosis was set to the date of the surgical therapy. Recurrence-free survival (RFS) was defined as the time from primary treatment to recurrence (within 5 years) or the date of death. The follow-up time for RFS was the time until diagnosis of relapse or until death or last contact in the respective treating departments. Patients were not considered for the recurrence analysis and classified as residual tumors when diagnosis was made within 6 months after primary surgery (± adjuvant therapy) with R1/Rx resection margins.

### 2.3. Immunohistochemistry

The detection of ERα expression was performed using immunohistochemistry (IHC) on tissue microarrays (TMA). In preparation, formalin-fixed paraffin-embedded (FFPE) tissue and the corresponding slides from patients with primary OSCC were collected from the archive of the Institute of Pathology, Charité–Universitätsmedizin Berlin, Germany. The tumor diagnoses were reviewed based on the current criteria of the WHO (WHO classification of head and neck tumors Lyon 2017). Hematoxylin–Eosin (HE) staining of all tumor blocks was performed, and representative areas were marked on these slides and the corresponding 2 µm-thick FFPE sections for IHC. New blocks were designed with one (or more) punches of the tumor, with a diameter of 1.0 mm.

The immunohistological staining was performed with the anti-ERα-antibody (clone SP1) from Ventana Medical Systems (Tucson, AZ, USA) as a ready-to-use antibody. The expression of ERα was quantified by light microscopy using the semiquantitative immunoreactive score (IRS) for estrogen receptor detection described by Remmele and Stegner [30]. However, in contrast to the original description, an IRS equal to or greater than 2 was considered positive, as described by other authors [31,32]. In the case of more than one stained tumor tissue per patient, arithmetic means were calculated.

### 2.4. Statistical Analysis

The data were collected in Microsoft Excel (Microsoft Corporation, WA, USA). Statistical analysis was performed using R Version 4.0.3 [33] and IBM SPSS Statistics Version 27 (IBM Corporation, NY, USA). Variables are shown as a frequency and percentage or mean value with standard deviation (SD). The 5-year OS and RFS were examined by the Kaplan–Meier method and log-rank test. Chi-square test was used to assess differences in categorical variables between ERα-positive and ERα-negative patients. To identify prognostic factors for OS and RFS, multiple cox regression with stepwise backward elimination was used. The initial model contained the variables ERα+ vs. ERα−, gender, age, pT stage, UICC stage, tumor localization, pN0 vs. pN1/pN2, G stage, and bony infiltration. At each step, the variable with the largest *p*-value was eliminated until all p-values were smaller than 0.1. The IRS of ERα remained in the model at all steps. The final model contained age, pT stage, and UICC stage, beside ERα+ vs. ERα− as independent variables. Hazard ratios for OS and RFS of ERα+ vs. ERα− are therefore adjusted with respect to age, pT stage, and UICC stage. All p-values are exploratory and are reported without adjustment for multiple testing. The level of statistical significance was set to *p* < 0.05.

## 3. Results

A total of 316 patients (111 females; 205 males) with a mean age of 61.3 years (range 27–96 years) diagnosed with primary OSCC were included in this study. Of these, 302 primary tumors and 52 primary lymph node metastases were analyzed for the expression of ERα. The cumulative 5-year overall survival (OS) was 61.9%, and recurrence-free survival (RFS) was 52.8%. The mean follow-up for survival was 63.0 months (SD ± 38.6). A total of 90 patients received adjuvant radio(chemo)therapy.

**Figure 1:**
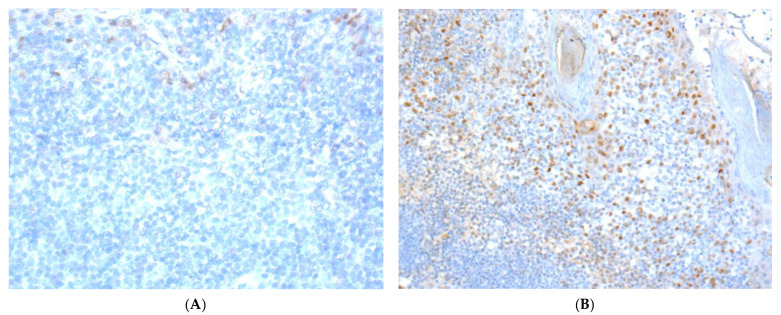
Immunohistochemical staining images of ERα in OSCC. (A) (40×): ERα-positive nuclear staining with weak intensity (Table 2; patient no. 3); (B) (20×): ERα-positive case with different intensities of nuclear staining (Table 2; patient no. 6; one out of three stained tumor tissue of this patient).

The mean immunoreactive score for ERα expression was 0.12 for all primary tumors (range 0–6) and 0.52 for all primary lymph node metastases (range 0–10). In 16 patients (5.1%; 6 females; 10 males), positive ERα expression (with an ERα equal to or greater than two) was found in the primary tumor (*n* = 11;11/302; 3.6%) or lymph node metastases (*n* = 5;5/52; 9.6%). Typical pictures of ERα IHC staining of primary tumors are shown in Figure 1.

The clinicopathological characteristics of the cohort are summarized in Table 1.

There was a significant difference (*p* = 0.006) in ERα positivity with regard to the localization of the primary tumor. The most frequent single localization was the lower jaw. ERα positivity in the primary tumor was significantly associated (*p* = 0.026) with UICC stage, with most of the cases being diagnosed in stage IV. Furthermore, there was a significantly (*p* = 0.049) higher rate of bone infiltration in ERα-positive patients in contrast to ERα-negative patients.

Table 2 summarizes the clinicopathological characteristics of all patients with an ERα-positive primary tumor.

Only for one of the patients with an ERα-positive primary tumor, the corresponding primary lymph node metastasis could be evaluated for ERα expression (patient no. 9; ERα IRS: 0.5).

**Figure 2:**
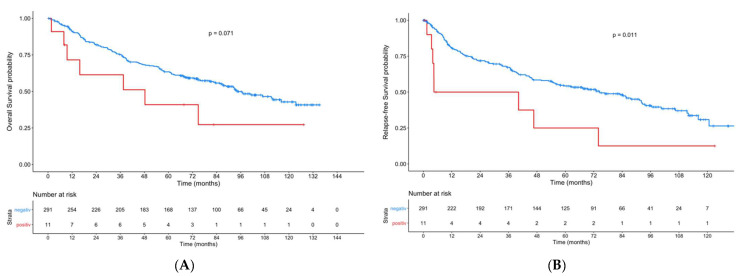
Kaplan–Meier curves showing overall survival (**A**) and recurrence-free-survival (**B**) for patients with ERα-positive primary tumors compared to patients with ERα-negative tumors.

Regarding the whole cohort, patients with ERα-positive primary tumors had a significant lower RFS (*p* = 0.011) compared to ERα-negative patients (Figure 2). For OS, a tendency was seen (*p* = 0.071).

In multivariate cox regression analysis, the ERα IRS of the primary tumors (dichotomized; ERα+ vs. ERα−) was not an independent risk factor for OS (HR = 1.039; 95%CI 0.455–2.371; *p* = 0.928) or RFS (HR = 1.352; 95%CI 0.617–2.962; *p* = 0.452). However, it was an independent risk factor for OS (HR = 4.230; 95%CI 1.616–11.076; *p* = 0.003) and RFS (HR = 12.390; 95%CI 4.073–37.693; *p* < 0.001) in the male cohort.

Table 3 summarizes the clinicopathological characteristics of all patients with an Erα-positive primary lymph node metastasis. ERα expression in four corresponding primary tumors was available. These were all negative.

**Figure 3:**
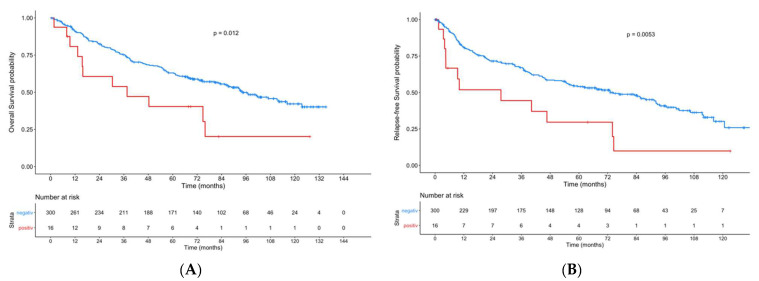
Kaplan–Meier curves showing overall survival (**A**) and recurrence-free-survival (**B**) in patients with an ERα-positive lymph node metastasis and/or primary tumor compared to patients with ERα-negative tumors.

When combining patients with an ERα-positive lymph node metastasis and/or primary tumor, we found significant lower OS (*p* = 0.012) and RFS (*p* = 0.0053) in the case of ERα positivity compared to ERα-negative patients (Figure 3).

**Figure 4:**
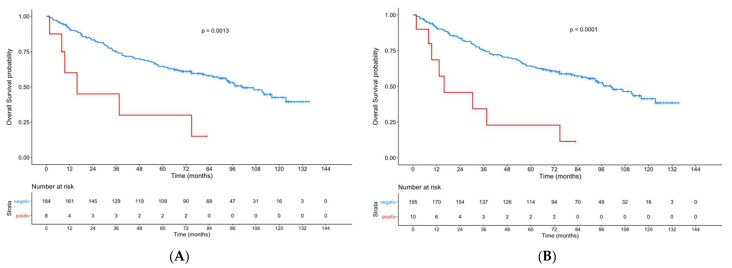
Kaplan–Meier curves showing the OS of male patients with an ERα-positive primary tumor (**A**) and the OS of male patients with ERα-positive lymph node metastasis and/or primary tumor (**B**) compared to patients with ERα-negative tumors.

Sub-group analysis in relation to gender revealed a highly significant influence on OS and RFS in males but not in females, for both the ERα-positive primary tumor cohort (males: *p* = 0.0013; *p* < 0.0001; females: *p* = 0.56; *p* = 0.89) and the ERα-positive primary tumor/primary lymph node metastasis cohort (males: *p* < 0.0001; *p* < 0.0001; females: *p* = 0.95; *p* = 0.96) (Figure 4).

**Figure 5:**
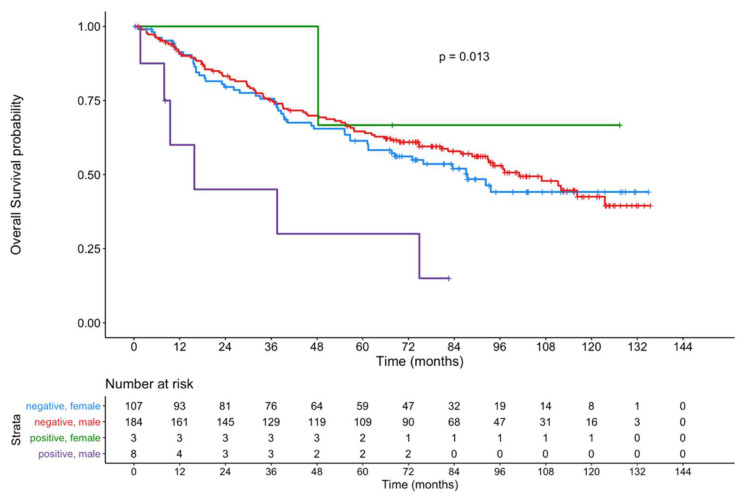
Kaplan–Meier curves showing the OS of male and female patients with an ERα-positive primary tumor compared to patients with ERα-negative tumors.

Figure 5 shows the OS of male and female patients with an ERα-positive primary tumor compared to patients with ERα-negative tumors. Within the limitations due to the small number of positive female patients, a significant difference (*p* = 0.013) was observed.

## 4. Discussion

The present study shows a prognostic significance of ERα in OSCC for a surgical cohort. Although the expression of ERα is rare, it is associated with a worse prognosis in comparison to ERα-negative patients. This effect was mainly observed in the male cohort, in whom a dramatic decrease in OS was observed. As successfully established in breast cancer, ERα-positive OSCC (male) patients might benefit from an ER-based therapeutic (adjuvant) approach in the future.

The estrogen receptors α and β are widely distributed in different cells and tissues of the human body [34]. In normal oral mucosa, immunohistochemical analyses showed ERβ but not ERα expression in males and females [26,35,36,37]. However, the nuclear expression of estrogen has been described in precursor lesions in the oral cavity [26]. Steroid receptor activation depends on the hormone binding to its cognate receptor, leading to dimerization and nuclear translocation. In the next step, this complex can interact with specific DNA sequences. Nevertheless, membrane ERα can also mediate some estrogen effects, like endothelial/vascular effects [38]. The cases investigated in the present study showed a nuclear expression pattern with variable intensity. Membrane staining could not be observed.

Information about the immunohistochemical expression of ERα and ERβ in OSCC primary tissue is rare and remains controversial. In most studies, including a study from our study group, ERβ was found to be the predominant sub-type in OSCC primary tissues [19,21,24,28].

The prevalence for ERα when evaluating (paraffin-embedded) OSCC tissue ranges from 0 to 13% [19,21,24,26,27,28]. A study published by Egloff et al. revealed an ERα positivity of 95% in 56 head and neck tumors that were evaluated (including 23 OSCC); however, detailed information only for the OSCC cohort was not reported [20]; therefore, a direct comparison is not possible. In the present study, the prevalence of ERα positivity in the primary tumor was 3.6%, which is comparably low than the findings of other studies. In this publication, we used a cut-off ≥2 for IRS, as described by other authors [31,32]. However, authors evaluating ERα in OSCC used different cut-off scores, which makes comparison difficult. Egloff et al. defined positivity as the (at least) weak expression of more than 10% of cells [20]. Marrocchio et al. considered neoplastic cells to be positive “when they showed nuclear reactivity” [24]. Positivity was defined as nuclear staining in ≥1% of tumor cells in a study published by Grimm et al. [26]. Kwon et al. established ERα positivity if more than 1% of the cancer cells showed nuclear staining [25]. Other studies lack any definition of positivity. 

The estrogen receptors are involved in the regulation of many complex physiological processes in humans. 

As mentioned, there are two different sub-types of estrogen receptors, which are widely distributed in different tissues. Perturbation of the expression of these receptors has been found in many different cancers and provides a possible target approach [6], as successfully established in breast carcinoma [7]. However, ER-mediated treatment strategies are being investigated in many different tissues that are not all considered to be primarily hormone-dependent [5,8,9,10]. Recently, an association between ERα expression and improved survival in OPSCC, which is another entity of the heterogenous group of head and neck cancers, has been found [25]. Kwon et al. analyzed the expression of ERα in 113 OPSCC patients. The authors observed that the expression of ERα was associated with better overall survival in both HPV+ (p16+/HPV+ OPSCC) and p16+ (p16+ OPSCC irrespective of HPV status) models.

To the best of our knowledge, Egloff et al. are the only group who demonstrated a prognostic influence of ERα in OSCC [20]. This study showed a significantly reduced progression-free survival in patients with high (nuclear) ERα (and EGFR) tumor levels. However, the study evaluated 56 head and neck tumors, with only 23 of these being OSCC. Due to the fact that distinct information only for OSCC has not been reported, it remains unclear whether ERα has a prognostic influence on OSCC. The present study showed a prognostic significance of ERα in OSCC. So far, the pathomechanism for the decrease in OS of ERα-positive patients remains unknown. The production of estrogens by inflammatory cells during chronic infection as a response to carcinogens might be an important hormonal source, as shown by Siegfried et. al. within lung cancer tissues [39].

The results of the present study clearly show that there is a significant association between ERα expression and prognosis in OSCC, especially in the male cohort. This might lead to the conclusion that ERα-positive OSCC patients might benefit from an ER-based therapeutic (adjuvant) approach. Various studies exist that prove the influence of ER agonists and antagonists on OSCC cell lines. Chang et al. observed a stimulated cell growth by incubation with estradiol, in contrast to tamoxifen or knockdown of ERα expression, which led to a reduced cell growth [17]. Ishida et al. showed that the treatment with tamoxifen, but not estradiol, caused apoptotic death in OSCC cell lines [21]. This was also observed by Nelson et al., whereas tamoxifen induced a significant growth inhibition in OSCC cell lines [22,23].

In contrast to other studies [19,21,24,26,27,28], one strength of this study is the analysis of ERα in a comparatively high number of patients in a homogenous cohort of OSCC. The main points of criticism are the retrospective data collection and the evaluation of ERα on tissue microarrays. The latter might lead to a problem whereby the evaluated tumor tissue does not reflect the expression of ERα within the entire tumor. Although the results of our exploratory study are very promising, the power of our statistical analyses is limited due to the low number of ERα-positive cases, especially in the female cohort. Further studies are necessary to confirm our results. An ER-based therapeutic (neo)adjuvant approach could be evaluated in a patient-derived xenograft mouse model.

## 5. Conclusions

The expression of ERα is rare in OSCC; however, it was associated with a dramatic decrease in OS in OSCC male patients. ERα expression was associated with an advanced tumor stage at initial diagnosis in our cohort. Further studies are necessary to confirm our results and evaluate the exact mechanism leading to this association. Hence, ERα-positive OSCC patients might benefit from an ER-based therapeutic (adjuvant) approach in the future.

## Figures and Tables

**Table 1:** Clinicopathological characteristics of the patient cohort in relation to the analysis of ERα expression in the primary tumor and lymph node metastases.

Variable	Primary Tumor (*n* = 302)			Primary Lymph Node Metastasis (*n* = 52)		
	ERα− (*n* = 291)	ERα+ (*n* = 11)	*p*	ERα− (*n* = 47)	ERα+ (*n* = 5)	*p*
Sex						
Female	107 (97.3%)	3 (2.7%)	0.521	10 (76.9%)	3 (23.1%)	0.057
Male	184 (95.8%)	8 (4.2%)		37 (94.9%)	2 (5.1%)	
Age group			0.504			0.121
≤49	45 (93.8%)	3 (6.3%)		10 (100%)	0 (0%)	
50–59	79 (98.8%)	1 (1.3%)		13 (81.3%)	3 (18.8%)	
60–69	100 (96.2%)	4 (3.8%)		18 (94.7%)	1 (5.3%)	
70–79	52 (94.5%)	3 (5.5%)		5 (100%)	0 (0%)	
80+	15 (100%)	0 (0%)		1 (50%)	1 (50%)	
Tumor localization			0.006			0.336
Floor of mouth	65 (98.5%)	1 (1.5%)		14 (82.4%)	3 (17.6%)	
Maxilla	13 (100%)	0 (0%)		-	-	
Mandible	27 (84.4%)	5 (15.6%)		4 (80%)	1 (20%)	
Cheek	13 (100%)	0 (0%)		-	-	
Tongue	80 (98.8%)	1 (1.2%)		8 (100%)	0 (0%)	
Multiple localizations	93 (95.9%)	4 (4.1%)		21 (95.5%)	1 (4.5%)	
History of Smoking			0.216			0.459
Yes	195 (95.1%)	10 (4.9%)		39 (90.7%)	4 (9.3%)	
No	67 (98.5%)	1 (1.5%)		4 (80.0%)	1 (20.0%)	
History of Alcohol			0.681			0.851
Yes	202 (96.2%)	8 (3.8%)		35 (89.7%)	4 (10.3%)	
No	57 (95.0%)	3 (5.0%)		7 (87.5%)	1 (12.5%)	
G-Stage			0.950			0.891
1	19 (95.0%)	1 (5.0%)		2 (100%)	0 (0%)	
2	215 (96.4%)	8 (3.6%)		28 (90.3%)	3 (9.7%)	
3	53 (96.4%)	2 (3.6%)		17 (89.5%)	2 (10.5%)	
pT			0.619			0.362
1	124 (97.6%)	3 (2.4%)		12 (80.0%)	3 (20.0%)	
2	109 (95.6%)	5 (4.4%)		16 (94.1%)	1 (5.9%)	
3	33 (97.1%)	1 (2.9%)		10 (100%)	0 (0%)	
4a	21 (91.3%)	2 (8.7%)		9 (90%)	1 (10%)	
x	4 (100%)	0 (0%)				
pN			0.092			-
N0	187 (97.4%)	5 (2.6%)		-	-	
N1/N2	82 (93.2%)	6 (6.8%)		47 (90.4%)	5 (9.6%)	
UICC			0.026			0.198
I	97 (100%)	0 (0%)		-	-	
II	75 (96.2%)	3 (3.8%)		-	-	
III	49 (96.1%)	2 (3.9%)		12 (100%)	0 (0%)	
IV	58 (90.6%)	6 (9.4%)		35 (87.5%)	5 (12.5%)	
Extracapsular spread (ECS)			0.204			0.704
N−	187 (97.4%)	5 (2.6%)		-	-	
N+ ECS−	47 (92.2%)	4 (7.8%)		23 (92.0%)	2 (8.0%)	
N+ ECS+	35 (94.6%)	2 (5.4%)		24 (88.9%)	3 (11.1%)	
Bony infiltration			0.049			0.913
no	231 (97.5%)	6 (2.5%)		27 (90.0%)	3 (10.0%)	
yes	60 (92.3%)	5 (7.7%)		20 (90.9%)	2 (9.1%)	

**Table 2:** Clinicopathological characteristics of all patients with an ERα-positive primary tumor.

No	Gender	Age	Localization	TNM	Grading	R-Status	ERα IRS	Smoking	Alcohol	Adjuvant Therapy	Survival (Months)	Time to Recurrence (Months)	Comment
1	male	66	Lower jaw	pT1pN1 cM0	G2	R0	2.00	+	+	−	75	None	
2	male	64	Floor of mouth	pT2pN1 cM0	G2	R0 cm *	2.00	+	−	+	8 (alive)	None	Lost to follow-up after 8 months.
3	male	49	Floor of mouth, tongue	pT2pN0 cM0	G2	R0	2.00	+	+	−	83 (alive)	Patient lost to clinical follow-up	Micrometastasis in one lymph node. The patient did not show up for regular clinical follow-up. However, the patient was still alive after 83 months.
4	male	39	Lower jaw	pT1pN2b cM0	G3	R0 *	6.00	+	+	(+)	8	3	Patient initially refused recommendation for adj. therapy. It was carried out 3 months after surgical therapy due to recurrence.
5	male	42	Tongue, floor of mouth	pT2pN2b cM0	G2	R0	2.00	+	+	+	9	4	
6	female	63	Lower jaw	pT4apN0 cM0	G2	R0; cm (3 mm)	3.33	−	−	+	68 (alive)	40	
7	male	57	Tongue	pT2pN0 cM0	G2	R0	2.00	+	−	−	16	4	
8	male	75	Lower jaw	pT1pN2b cM0	G2	R0 *	3.00	+	+	(+)	38	4	Adjuvant therapy was carried out after nodal recurrence about 4 months after surgical therapy. Initially, it could not be performed due to a strongly reduced general condition.
9	female	67	Lower jaw, floor of mouth	pT3pN2c cM0	G2	R0; cm *	3.00	+	+	+	127 (alive)	None	ERα IRS of corresponding lymph node metastasis: 0.5
10	female	71	Lower jaw	pT2pN0 cM0	G1	R0 *	2.00	+	+	−	48	47	
11	male	70	Lower jaw, floor of mouth	pT4apN0 cM1	G3	R0; cm (4 mm)	2.00	+	+	−	2	None	Patient died within the inpatient stay after surgical therapy 2 months after first diagnosis.

* no information on exact safety margin; cm: close margin.

**Table 3:** Clinicopathological characteristics of all patients with an ERα-positive lymph node metastasis.

No	Gender	Age	ERα IRS		Localization	Grading	R-Status	pTNM	Smoking	Alcohol	Adjuvant Therapy	Survival (Months)	Time to Recurrence (Months)	Comment
			Lymph Node Metastasis	Primary Tumor										
1	female	53	5.00	1.00	Floor of mouth	G2	R0; cm (4 mm)	pT1 pN2b cM0	+	+	+	69 (alive)	none	
2	male	51	3.00	0	Floor of mouth	G2	R0; cm**	pT1 pN2c cM0	+	+	+	30	none	
3	male	51	3.75	*	Lower jaw	G3	R0 **	pT2 pN2b cM0	+	+	+	13	none	Adj. therapy was delayed due to a strongly reduced general condition. It was carried out 2.5 months after surgical approach.
4	female	62	10.00	1.33	Floor of mouth, lower jaw, tongue	G2	R1	pT4a pN2c cM0	+	+	+	16	10	
5	female	80	5.00	0	Floor of mouth	G3	R0; cm (4 mm)	pT1 pN2b cM0	−	−	−	76	Patient lost to clinical follow-up	Patient refused recommendation for adj. therapy. The patient did not show up for regular clinical follow-up. However, the patient died 76 months after first diagnosis.

* Not available; ** no information on exact safety margin; cm: close margin.

## Data Availability

The data presented in this study are available on request from the corresponding author.
